# Supplementary material for: Multi-cohort study identifies social determinants of systemic inflammation over the life course
Source: Nat Commun. 2019 Feb 15;10:773. doi: 10.1038/s41467-019-08732-x (PMC6377676; doi:10.1038/s41467-019-08732-x)
Supplement: Supplementary file 1 — Supplementary Information [file 41467_2019_8732_MOESM1_ESM.pdf]

Supplementary Information for

Multi-cohort study identifies social determinants of systemic inflammation over the life course

Eloïse Berger[1,¤], Raphaële Castagné[1,¤], Marc Chadeau-Hyam[2], Murielle Bochud[3], Angelo d'Errico[4], Martina Gandini[5], Maryam Karimi[2], Mika Kivimäki[5,6], Vittorio Krogh[7], Michael Marmot[5], Salvatore Panico[8], Martin Preisig[9], Fulvio Ricceri[4], Carlotta Sacerdote[10], Andrew Steptoe[5], Silvia Stringhini[9], Rosario Tumino[11], Paolo Vineis[2,12,µ], Cyrille Delpierre[1,µ], Michelle Kelly-Irving[1,µ,*] ; LIFEPATH consortium

[*]**Address correspondence to:**

Michelle Kelly-Irving

LEASP, UMR 1027, Inserm-Université Toulouse III Paul Sabatier

Faculty of Medicine Purpan, 37 allées Jules Guesde, 31000 Toulouse

E-mail: michelle.kelly@inserm.fr

This PDF file includes:

Supplementary text
Supplementary Figure 1-3
Supplementary Tables 1-14

Supplementary note 1

The Lifepath consortium consists of:

Harri Alenius, Mauricio Avendano, Valeria Baltar, Mel Bartley, Henrique Barros, , Cristian Carmeli, Luca Carra, Françoise Clavel-Chapelon, Giuseppe Costa, Emilie Courtin, Angela Donkin, Angelo D'Errico, Pierre-Antoine Dugue, Paul Elliott, Giovanni Fiorito, Silvia Fraga, Valérie Garès, Martina Gandini, Graham Giles, Marcel Goldberg, Dario Greco, Allison Hodge, Piia Karisola, Jessica Laine, Thierry Lang, Richard Layte, Benoit Lepage, Johan Mackenbach, Carlos de Mestral, Cathal McCrory, Roger Milne, Peter Muennig, Wilma Nusselder, Dusan Petrovic, Silvia Polidoro, Martin Preisig, Olli Raitakari, Ana Isabel Ribeiro, Fulvio Ricceri, Erica Reinhard, Oliver Robinson, Jose Rubio Valverde, Roberto Satolli, Gianluca Severi, Martin J Shipley, Joannie Tieulent, Salvatore Vaccarella, Anne-Claire Vergnaud, Peter Vollenweider, Marie Zins

Supplementary note 2

CRP measurements at follow-up

In the CoLaus study, high-sensitivity C-reactive protein (hs-CRP, mg/L) was assessed by immunoassay and latex HS (IMMULITE 1000-High, Diagnostic Products Corporation, Los Angeles, CA, USA). In Whiteall, CRP was measured using a high-sensitivity immunonephelometric assay in a BN ProSpec nephelometer (Dade Behring, Milton Keynes, UK). In ELSA, circulating hs-CRP was assessed using the N latex CRP mono immunoassay on the Dade Behring Nephelometer II Analyzer.

Supplementary note 3

Regression coefficient attenuation:

We calculated the percentage attenuation in the β coefficient for BMI and life-course SEP in the relevant models using the following formula:

$$\Delta = 100 \times \frac{\beta_{Model\ 1} - \beta_{Model\ 1+BMI}}{\beta_{Model\ 1}}$$

Supplementary Table 1. Multiple regression analyses in men of (A) father's occupational position in 4 cohorts (B) participant's educational attainment and (C) participant's last occupation with CRP at baseline in 6 cohorts from the Lifepath project.

		**(A) Father's occupational position (N=7798)†**		**(B) Participant's educational attainment (N=12809)‡**		**(C) Participant's last occupation (N=12809)‡**	
	Reference	Advantaged (8.2%)		High (27.2%)		Advantaged (41.6%)	
	Level	Middle (27.8%)	Disadvantaged (64.0%)	Medium (19.9%)	Low (53.0%)	Middle (30.0%)	Disadvantaged (28.5%)
**Model 1***	β (95% CI)	0.003 (-0.10; 0.10)	0.12 (0.03; 0.22)	0.15 (0.08; 0.21)	0.28 (0.18; 0.37)	0.12 (0.07; 0.17)	0.21 (0.09; 0.33)
	P-value	0.958	0.011	<0.001	<0.001	<0.001	0.001
	I^2	0	0	6.19	69.3	10.8	77.9
	P_H	0.421	0.859	0.243	0.014	0.362	0.001
**Model 1* + Alcohol**	β (95% CI)	-0.001 (-0.10; 0.10)	0.12 (0.02; 0.21)	0.14 (0.08; 0.20)	0.27 (0.19; 0.35)	0.12 (0.07; 0.16)	0.20 (0.07; 0.32)
	P-val	0.989	0.014	<0.001	<0.001	<0.001	0.002
	I^2	0.001	0	0.023	59.1	0	79.2
	P_H	0.377	0.939	0.302	0.040	0.539	0.001
**Model 1* + Smoking**	β (95% CI)	0.004 (-0.10; 0.10)	0.11 (0.02; 0.20)	0.13 (0.06; 0.20)	0.22 (0.13; 0.32)	0.10 (0.04; 0.15)	0.16 (0.04; 0.27)
	P-value	0.936	0.021	<0.001	<0.001	<0.001	0.006
	I^2	0	0	20.1	72.3	8.6	73.3
	P_H	0.439	0.922	0.228	0.008	0.522	0.001
**Model 1* + BMI**	β (95% CI)	-0.02 (-0.12; 0.07)	0.07 (-0.02; 0.16)	0.10 (0.04; 0.16)	0.20 (0.10; 0.30)	0.11 (0.06; 0.16)	0.17 (0.07; 0.28)
	P-value	0.642	0.139	0.001	<0.001	<0.001	0.001
	I^2	0.005	0	2.39	74.7	2.9	71.5
	P_H	0.254	0.937	0.333	0.006	0.474	0.006
**Model 1*+ Sedentary**	β (95% CI)	0.05 (-0.08; 0.17)	0.10 (-0.01; 0.22)	0.13 (0.07; 0.19)	0.23 (0.16; 0.30)	0.11 (0.06; 0.16)	0.21 (0.12; 0.30)
	P-value	0.460	0.08	<0.001	<0.001	<0.001	<0.001
	I^2	0	0	0	35.1	0	43.3
	P_H	0.485	0.965	0.413	0.165	0.415	0.090
**Model 2¤**	β (95% CI)	-0.01 (-0.12; 0.10)	0.05 (-0.04; 0.14)	0.08 (0.03; 0.14)	0.13 (0.05; 0.21)	0.07 (0.02; 0.11)	0.09 (0.01; 0.17)
	P-value	0.821	0.236	0.005	0.001	0.005	0.025
	I^2	16.3	0	0	57.5	0	48.3
	P_H	0.230	0.957	0.501	0.049	0.890	0.075

†Except for model 1 + sedentary where N = 4975

‡Except for model 1 + sedentary where N = 9986

*Model 1 adjusted for age

¤Model 2 controlled for age and additionally alcohol, smoking, BMI and sedentary

Abbreviations: CI, confidence interval; I^2, percentage of between study heterogeneity; P_H, P-value of heterogeneity test; BMI, body mass index.

Supplementary Table 2. Multiple regression analyses in women of (A) father's occupational position in 4 cohorts (B) participant's educational attainment and (C) participant's last occupation with CRP at baseline in 6 cohorts from the Lifepath project.

		**(A) Father's occupational position (N=5280)[†]**		**(B) Participant's educational attainment (N=10199)[‡]**		**(C) Participant's last occupation (N=10199)[‡]**	
	Reference	Advantaged (7.9%)		High (19.5%)		Advantaged (19.1%)	
	Level	Middle (24.9%)	Disadvantaged (67.2%)	Medium (15.5%)	Low (65.0%)	Middle (39.1%)	Disadvantaged (41.8%)
**Model 1[*]**	β (95% CI)	0.16 (-0.04; 0.37)	0.28 (0.16; 0.40)	0.15 (0.02; 0.27)	0.33 (0.21; 0.45)	0.10 (-0.03; 0.24)	0.31 (0.20; 0.41)
	P-value	0.121	<0.001	0.021	<0.001	0.136	<0.001
	I^2	57.5	0	55.2	67.9	71.3	51.3
	P_H	0.073	0.589	0.050	0.009	0.004	0.104
**Model 1[*] + Alcohol**	β (95% CI)	0.15 (-0.06; 0.36)	0.26 (0.13; 0.38)	0.14 (0.02; 0.26)	0.31 (0.19; 0.42)	0.09 (-0.05; 0.22)	0.26 (0.16; 0.36)
	P-val	0.169	<0.001	0.025	<0.001	0.200	<0.001
	I^2	60.2	0	51.9	67.8	69.6	44.0
	P_H	0.060	0.501	0.071	0.010	0.007	0.176
**Model 1[*] + Smoking**	β (95% CI)	0.17 (-0.04; 0.37)	0.27 (0.15; 0.39)	0.14 (0.02; 0.27)	0.31 (0.20; 0.43)	0.10 (-0.04; 0.24)	0.29 (0.18; 0.40)
	P-value	0.120	<0.001	0.026	<0.001	0.154	<0.001
	I^2	57.6	0	53.8	66.9	71.3	53.7
	P_H	0.072	0.556	0.060	0.012	0.004	0.086
**Model 1[*] + BMI**	β (95% CI)	0.08 (-0.07; 0.24)	0.15 (0.03; 0.26)	0.09 (-0.03; 0.20)	0.19 (0.07; 0.30)	0.06 (-0.03; 0.15)	0.19 (0.13; 0.25)
	P-value	0.304	0.013	0.128	0.001	0.213	<0.001
	I^2	35.7	0	52.8	71.0	49.5	0
	P_H	0.204	0.458	0.069	0.003	0.102	0.580
**Model 1[*]+ Sedentary**	β (95% CI)	0.23 (0.06; 0.39)	0.26 (0.11; 0.41)	0.14 (0.001; 0.27)	0.28 (0.16; 0.40)	0.15 (0.03; 0.27)	0.31 (0.22; 0.41)
	P-value	0.006	0.001	0.049	<0.001	0.014	<0.001
	I^2	3.0	0.003	55.2	61.5	46.7	20.9
	P_H	0.424	0.343	0.070	0.037	0.112	0.468
**Model 2[¤]**	β (95% CI)	0.07 (-0.09; 0.22)	0.12 (0.002; 0.24)	0.07 (-0.04; 0.18)	0.14 (0.04; 0.25)	0.04 (-0.05; 0.13)	0.13 (0.07; 0.19)
	P-value	0.386	0.046	0.208	0.009	0.363	<0.001
	I^2	32.7	4.57	47.0	67.6	44.4	0
	P_H	0.222	0.362	0.108	0.010	0.140	0.786

[†]Except for model 1 + sedentary where N = 2794

[‡]Except for model 1 + sedentary where N = 7713

[*]Model 1 adjusted for age

[¤]Model 2 controlled for age and additionally alcohol, smoking, BMI and sedentary

Abbreviations: CI, confidence interval; I^2, percentage of between study heterogeneity; P_H, P-value of heterogeneity test; BMI, body mass index.

Supplementary Table 3. Life course multiple regression analyses in men of SEP with CRP at baseline in a random effect meta-analytical framework from 6 cohorts from the Lifepath.

		Model A*					Model Bµ				
	**Level**	**β**	**95% CI**	**P-value**	I^2	P_H	**β**	**95% CI**	**P-value**	I^2	P_H
**Father's occupation**	Middle	0.003	(-0.10; 0.10)	0.958	0	0.421	-0.03	(-0.13; 0.07)	0.581	0	0.409
	Disadvantaged	0.12	(0.03; 0.22)	0.011	0	0.859	0.05	(-0.04; 0.15)	0.279	0	0.976
**Educational level**	Medium	-	-	-	-	-	0.11	(0.01; 0.20)	0.033	14.8	0.257
	Low	-	-	-	-	-	0.21	(0.08; 0.35)	0.002	66.4	0.044
**Last occupation**	Middle	-	-	-	-	-	-	-	-	-	-
	Disadvantaged	-	-	-	-	-	-	-	-	-	-
		**Model C$**					**Model D#**				
	**Level**	**β**	**95% CI**	**P-value**	I^2	P_H	**β**	**95% CI**	**P-value**	I^2	P_H
**Father's occupation**	Middle	-0.004	(-0.11; 0.10)	0.938	0.006	0.388	-0.03	(-0.13; 0.07)	0.585	0	0.407
	Disadvantaged	0.10	(0.01; 0.20)	0.032	0	0.832	0.05	(-0.04; 0.15)	0.280	0	0.943
**Educational level**	Medium	-	-	-	-	-	0.10	(0.02; 0.18)	0.014	0.064	0.293
	Low	-	-	-	-	-	0.22	(0.07; 0.37)	0.004	68.3	0.018
**Last occupation**	Middle	0.09	(0.02; 0.15)	0.011	0.545	0.501	0.05	(-0.02; 0.12)	0.139	0.153	0.511
	Disadvantaged	0.11	(-0.08; 0.30)	0.259	77.8	0.005	0.01	(-0.22; 0.25)	0.913	83.2	0.001
		**Fully adjusted¤**									
	**Level**	**β**	**95% CI**	**P-value**	I^2	P_H					
**Father's occupation**	Middle	-0.02	(-0.14; 0.11)	0.810	31.3	0.197					
	Disadvantaged	0.03	(-0.06; 0.12)	0.532	0	0.804					
**Educational level**	Medium	0.04	(-0.03; 0.12)	0.275	0	0.475					
	Low	0.10	(-0.05; 0.24)	0.184	69.2	0.025					
**Last occupation**	Middle	0.02	(-0.04; 0.08)	0.528	0	0.971					
	Disadvantaged	-0.02	(-0.15; 0.12)	0.778	52.3	0.097					

*Model A adjusted for age, father's occupational position

µModel B adjusted for age, father's occupational position and participant's educational attainment

$Model C adjusted for age, father's occupational position and participant's last occupation

#Model D adjusted for age, father's occupational position, participant's educational attainment and participant's last occupation

¤Fully adjusted model controlled age, father's occupational position, participant's educational attainment, participant's last occupation and additionally alcohol, smoking, BMI and sedentary

Abbreviations: CI, confidence interval; I^2, heterogeneity; P_H, P-value of heterogeneity test; BMI, body mass index.

Supplementary Table 4. Life course multiple regression analyses in women of SEP with CRP at baseline in a random effect meta-analytical framework from 6 cohorts from the Lifepath.

		**Model A[*]**					**Model Bµ**				
	**Level**	**β**	**95% CI**	**P-value**	**I^2**	**P_H**	**β**	**95% CI**	**P-value**	**I^2**	**P_H**
**Father's occupation**	Middle	0.16	(-0.04; 0.37)	0.121	57.5	0.073	0.10	(-0.09; 0.30)	0.296	50.3	0.113
	Disadvantaged	0.28	(0.16; 0.40)	<0.001	0	0.589	0.18	(0.05; 0.31)	0.006	0.023	0.493
**Educational level**	Medium						0.11	(-0.06; 0.28)	0.202	47.4	0.136
	Low						0.28	(0.10; 0.46)	0.002	66.0	0.029
**Last occupation**	Middle						-	-	-	-	-
	Disadvantaged						-	-	-	-	-
		**Model C[$]**					**Model D[#]**				
	**Level**	**β**	**95% CI**	**P-value**	**I^2**	**P_H**	**β**	**95% CI**	**P-value**	**I^2**	**P_H**
**Father's occupation**	Middle	0.13	(-0.07; 0.33)	0.192	54.0	0.093	0.10	(-0.10; 0.29)	0.329	49.5	0.118
	Disadvantaged	0.22	(0.09; 0.36)	0.001	10.9	0.296	0.16	(0.04; 0.29)	0.013	0.032	0.497
**Educational level**	Medium	-	-	-	-	-	0.11	(-0.11; 0.32)	0.327	62.9	0.037
	Low	-	-	-	-	-	0.24	(-0.03; 0.51)	0.076	82.0	0.001
**Last occupation**	Middle	0.05	(-0.15; 0.25)	0.631	67.8	0.010	0.01	(-0.24; 0.26)	0.963	77.5	0.001
	Disadvantaged	0.22	(0.06; 0.39)	0.009	51.5	0.102	0.14	(-0.10; 0.38)	0.250	70.7	0.007
		**Fully adjusted[¤]**									
	**Level**	**β**	**95% CI**	**P-value**	**I^2**	**P_H**					
**Father's occupation**	Middle	0.04	(-0.10; 0.18)	0.585	22.1	0.295					
	Disadvantaged	0.08	(-0.04; 0.20)	0.193	0.021	0.442					
**Educational level**	Medium	0.06	(-0.15; 0.26)	0.586	64.3	0.033					
	Low	0.13	(-0.10; 0.35)	0.263	77.3	0.004					
**Last occupation**	Middle	-0.003	(-0.20; 0.19)	0.976	67.6	0.015					
	Disadvantaged	0.06	(-0.11; 0.23)	0.498	50.0	0.125					

*Model A adjusted for age, father’s occupational position

µModel B adjusted for age, father’s occupational position and participant's educational attainment

[$]Model C adjusted for age, father’s occupational position and participant's last occupation

[#]Model D adjusted for age, father’s occupational position, participant's educational attainment and participant's last occupation

[¤]Fully adjusted model controlled age, father’s occupational position, participant's educational attainment, participant's last occupation and additionally alcohol, smoking, BMI and sedentary

Abbreviations: CI, confidence interval; I2, heterogeneity; PH, P-value of heterogeneity test; BMI, body mass index.

Supplementary Figure 1. Forest plot of regression coefficient [95% confidence interval] of the association between father's occupational position and CRP concentration at baseline by cohort, in random effect meta-analysis framework for the total population for Model 1 (M1) and after adjustment on behavioural and lifestyle intermediate factors.

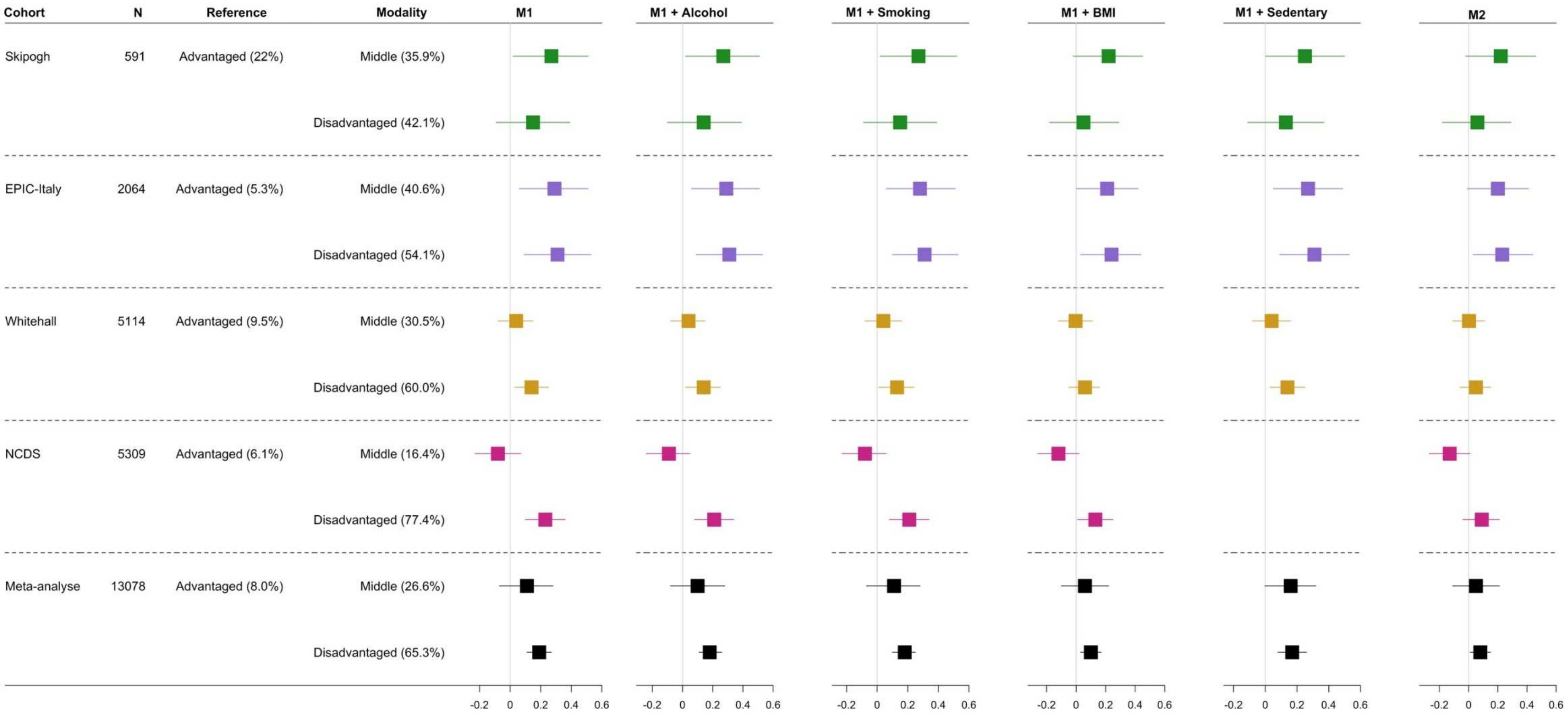

M1 is adjusted for age and sex (except in NCDS, sex only).
Meta-analyses for M1 + sedentary includes 7769 participants in all population.

Supplementary Table 5. Multiple regression analyses of father occupational position with CRP at baseline in 4 cohorts from the Lifepath project.

			**Model 1**			**Model 1 + Alcohol**			**Model 1 + Smoking**			**Model 1 + BMI**			**Model 1 + Sedentary**			**Model 2**		
	Ref.	Level	β	95% CI	P-value	β	95% CI	P-value	β	95% CI	P-value	β	95% CI	P-value	β	95% CI	P-value	β	95% CI	P-value
**Skipogh (N=591)**	Advantaged (22%)	Middle (35.9%)	0.27	(0.02; 0.51)	0.034	0.27	(0.02; 0.51)	0.036	0.27	(0.02; 0.52)	0.034	0.22	(-0.02; 0.45)	0.071	0.25	(0.002; 0.50)	0.049	0.22	(-0.02; 0.46)	0.071
		Disadvantaged (42.1%)	0.15	(-0.09; 0.39)	0.227	0.14	(-0.10; 0.39)	0.240	0.15	(-0.09; 0.39)	0.227	0.05	(-0.18; 0.29)	0.659	0.13	(-0.11; 0.37)	0.305	0.06	(-0.18; 0.29)	0.647
**EPIC-Italy (N=2064)**	Advantaged (5.3%)	Middle (40.6%)	0.29	(0.06; 0.51)	0.012	0.29	(0.06; 0.51)	0.012	0.28	(0.06; 0.51)	0.011	0.21	(0.003; 0.42)	0.047	0.27	(0.05; 0.49)	0.017	0.20	(-0.01; 0.41)	0.057
		Disadvantaged (54.1%)	0.31	(0.09; 0.53)	0.005	0.31	(0.09; 0.53)	0.005	0.31	(0.10; 0.53)	0.005	0.24	(0.03; 0.44)	0.026	0.31	(0.09; 0.53)	0.006	0.23	(0.03; 0.44)	0.026
**Whitehall (N=5114)**	Advantaged (9.5%)	Middle (30.5%)	0.04	(-0.08; 0.15)	0.564	0.04	(-0.08; 0.15)	0.565	0.04	(-0.08; 0.16)	0.527	-0.004	(-0.12; 0.11)	0.940	0.04	(-0.08; 0.16)	0.473	0.002	(-0.11; 0.11)	0.965
		Disadvantaged (60.0%)	0.14	(0.03; 0.25)	0.016	0.14	(0.02; 0.25)	0.017	0.13	(0.01; 0.24)	0.027	0.06	(-0.05; 0.16)	0.280	0.14	(0.03; 0.25)	0.013	0.05	(-0.06; 0.15)	0.379
**NCDS (N=5309)**	Advantaged (6.1%)	Middle (16.4%)	-0.08	(-0.23; 0.07)	0.273	-0.09	(-0.24; 0.05)	0.208	-0.08	(-0.23; 0.06)	0.261	-0.12	(-0.26; 0.02)	0.095	-			-0.13	(-0.27; 0.01)	0.067
		Disadvantaged (77.4%)	0.23	(0.10; 0.36)	0.001	0.21	(0.08; 0.34)	0.002	0.21	(0.08; 0.34)	0.002	0.13	(0.01; 0.25)	0.039				0.09	(-0.04; 0.21)	0.161

Model 1 is adjusted for age and sex (except for NCDS, sex only)
Meta-analysis with random effect on a total of 13,078 participants (except for model 1 + sedentary where N = 7,769)

Supplementary Figure 2. Forest plot of regression coefficient [95% confidence interval] of the association between educational attainment and CRP concentration at baseline by cohort, in random effect meta-analysis framework for the total population for Model 1 (M1) and after adjustment on behavioural and lifestyle intermediate factors.

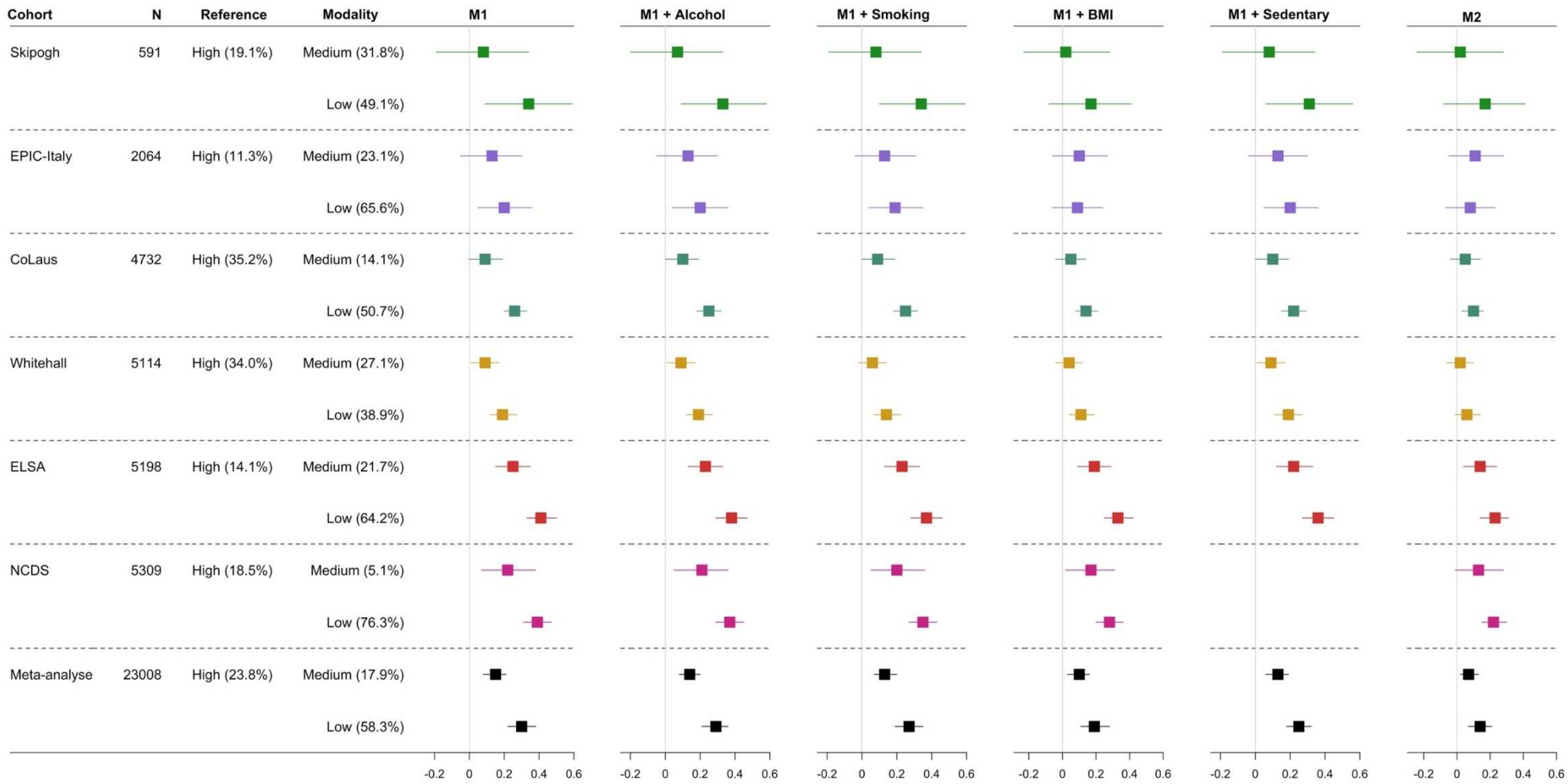

M1 is adjusted for age and sex (except in NCDS, sex only).
Meta-analyses on M1 + sedentary includes 17699 participants in all population.

Supplementary Table 6. Multiple regression analyses of participant’s educational attainment with CRP at baseline in 6 cohorts from the Lifepath project.

			Model 1			Model 1 + Alcohol			Model 1 + Smoking			Model 1 + BMI			Model 1 + Sedentary			Model 2		
	Ref.	Level	β	95% CI	P-value	β	95% CI	P-value	β	95% CI	P-value	β	95% CI	P-value	β	95% CI	P-value	β	95% CI	P-value
**Skipogh (N=591)**	High (19.1%)	Medium (31.8%)	0.08	(-0.19; 0.34)	0.578	0.07	(-0.20; 0.33)	0.619	0.08	(-0.19; 0.34)	0.578	0.02	(-0.23; 0.28)	0.865	0.08	(-0.19; 0.34)	0.575	0.02	(-0.24; 0.28)	0.874
		Low (49.1%)	0.34	(0.09; 0.59)	0.007	0.33	(0.09; 0.58)	0.009	0.34	(0.10; 0.59)	0.007	0.17	(-0.08; 0.41)	0.184	0.31	(0.06; 0.56)	0.014	0.17	(-0.08; 0.41)	0.190
**EPIC-Italy (N=2064)**	High (11.3%)	Medium(23.1%)	0.13	(-0.05; 0.30)	0.150	0.1à3	(-0.05; 0.30)	0.154	0.13	(-0.04; 0.31)	0.135	0.10	(-0.06; 0.27)	0.215	0.13	(-0.04; 0.30)	0.144	0.11	(-0.05; 0.28)	0.182
		Low (65.6%)	0.20	(0.05; 0.36)	0.011	0.2	(0.04; 0.36)	0.012	0.19	(0.04; 0.35)	0.014	0.09	(-0.06; 0.24)	0.240	0.20	(0.05; 0.36)	0.012	0.08	(-0.07; 0.23)	0.29
**CoLaus (N=4732)**	High (35.2%)	Medium (14.1%)	0.09	(-0.003; 0.19)	0.058	0.10	(0; 0.19)	0.051	0.09	(-0.001; 0.19)	0.053	0.05	(-0.04; 0.14)	0.293	0.10	(0; 0.19)	0.05	0.05	(-0.04; 0.14)	0.245
		Low (50.7%)	0.26	(0.20; 0.33)	<0.001	0.25	(0.18; 0.32)	<0.001	0.25	(0.18; 0.32)	<0.001	0.14	(0.08; 0.21)	<0.001	0.22	(0.15; 0.29)	<0.001	0.10	(0.03; 0.16)	0.003
**Whitehall (N=5114)**	High (34.0%)	Medium (27.1%)	0.09	(0.01; 0.17)	0.034	0.09	(0.01; 0.17)	0.032	0.06	(-0.02; 0.14)	0.135	0.04	(-0.04; 0.12)	0.317	0.09	(0.01; 0.17)	0.031	0.02	(-0.06; 0.10)	0.65
		Low (38.9%)	0.20	(0.12; 0.27)	<0.001	0.19	(0.12; 0.27)	<0.001	0.14	(0.07; 0.22)	<0.001	0.11	(0.04; 0.19)	0.002	0.19	(0.11; 0.27)	<0.001	0.06	(-0.01; 0.14)	0.099
**ELSA (N=5198)**	High (14.1%)	Medium (21.7%)	0.25	(0.15; 0.35)	<0.001	0.23	(0.13; 0.33)	<0.001	0.23	(0.13; 0.33)	<0.001	0.19	(0.09; 0.29)	<0.001	0.22	(0.12; 0.33)	<0.001	0.14	(0.04; 0.24)	0.004
		Low (64.2%)	0.41	(0.33; 0.50)	<0.001	0.38	(0.29; 0.47)	<0.001	0.37	(0.28; 0.46)	<0.001	0.33	(0.25; 0.42)	<0.001	0.36	(0.27; 0.45)	<0.001	0.23	(0.14; 0.31)	<0.001
**NCDS (N=5309)**	High (18.5%)	Medium (5.1%)	0.22	(0.07; 0.38)	0.005	0.21	(0.05; 0.36)	0.009	0.20	(0.05; 0.36)	0.011	0.17	(0.02; 0.31)	0.027	-			0.13	(-0.01; 0.28)	0.075
		Low (N=76.3%)	0.39	(0.31; 0.47)	<0.001	0.37	(0.29; 0.45)	<0.001	0.35	(0.27; 0.43)	<0.001	0.28	(0.20; 0.36)	<0.001				0.22	(0.15; 0.30)	<0.001

Model 1 is adjusted for age and sex (except for NCDS, sex only)
Meta-analysis with random effect on a total of 23 008 participants (except for model 1 + sedentary where N = 17699)

Supplementary Figure 3. Forest plot of regression coefficient [95% confidence interval] of the association between participant's last occupation and CRP concentration at baseline by cohort, in random effect meta-analysis framework for the total population for Model 1 (M1) and after adjustment on behavioural and lifestyle intermediate factors.

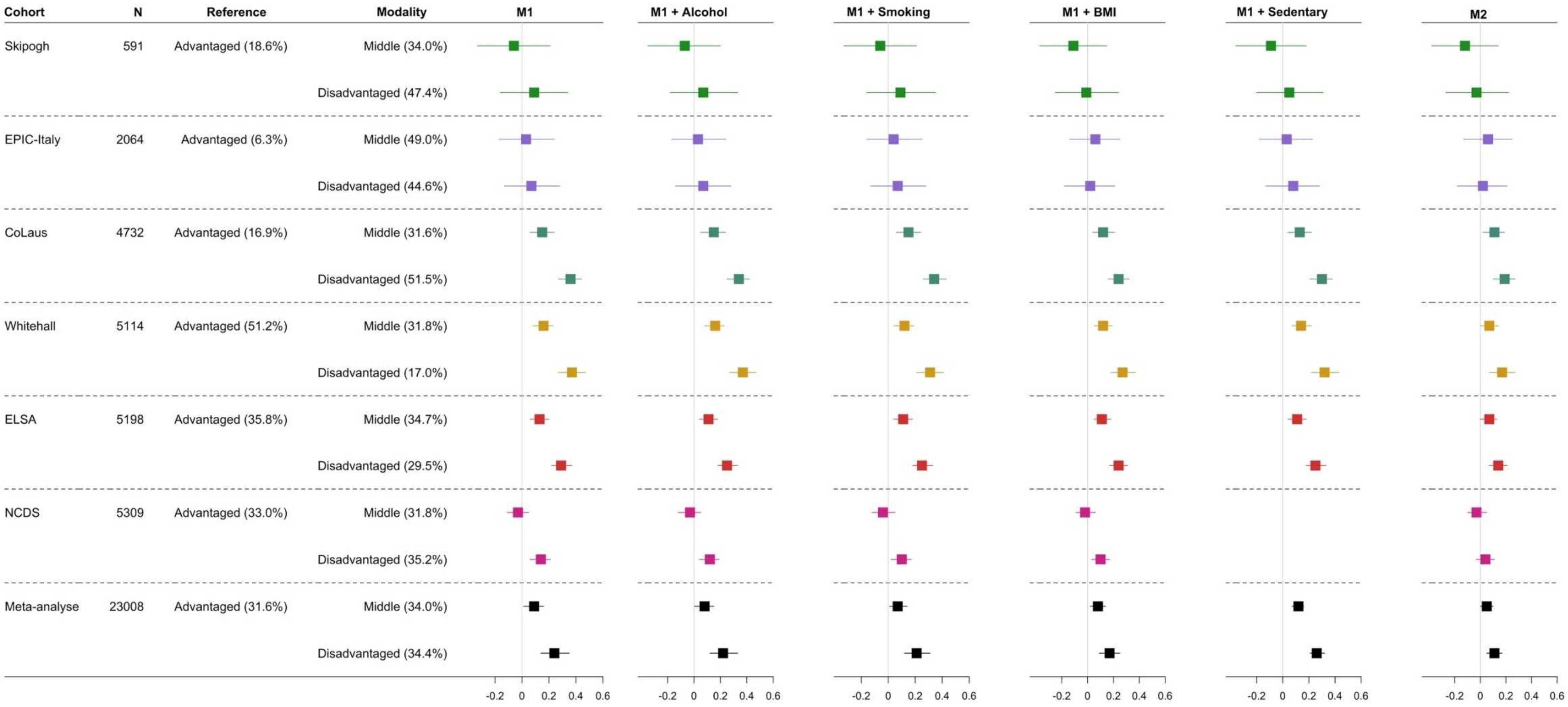

M1 is adjusted for age and sex (except in NCDS, sex only).
Meta-analyses on M1 + sedentary includes 17699 participants in all population.

Supplementary Table 7. Multiple regression analyses of participant's last occupation with CRP at baseline in 6 cohorts from the Lifepath project.

			Model 1			Model 1 + Alcohol			Model 1 + Smoking			Model 1 + BMI			Model 1 + Sedentary			Model 2		
	Ref.	Level	β	95% CI	P-value	β	95% CI	P-value	β	95% CI	P-value	β	95% CI	P-value	β	95% CI	P-value	β	95% CI	P-value
**Skipogh (N=591)**	Advantaged (18.6%)	Middle (34.0%)	-0.06	(-0.33; 0.21)	0.667	-0.07	(-0.35; 0.20)	0.593	-0.06	(-0.33; 0.21)	0.672	-0.11	(-0.37; 0.15)	0.421	-0.09	(-0.36; 0.18)	0.534	-0.12	(-0.38; 0.14)	0.359
		Disadvantaged (47.4%)	0.09	(-0.16; 0.34)	0.474	0.07	(-0.18; 0.33)	0.567	0.09	(-0.16; 0.35)	0.47	-0.01	(-0.25; 0.24)	0.970	0.05	(-0.20; 0.31)	0.679	-0.03	(-0.27; 0.22)	0.84
**EPIC-Italy (N=2064)**	Advantaged (6.3%)	Middle (49.0%)	0.03	(-0.17; 0.24)	0.744	0.03	(-0.17; 0.24)	0.742	0.04	(-0.16; 0.25)	0.676	0.06	(-0.14; 0.25)	0.579	0.03	(-0.18; 0.23)	0.784	0.06	(-0.13; 0.25)	0.528
		Disadvantaged (44.6%)	0.07	(-0.13; 0.28)	0.500	0.07	(-0.14; 0.28)	0.501	0.07	(-0.13; 0.28)	0.495	0.02	(-0.18; 0.21)	0.864	0.08	(-0.13; 0.28)	0.467	0.02	(-0.18; 0.21)	0.857
**CoLaus (N=4732)**	Advantaged (16.9%)	Middle (31.6%)	0.15	(0.06; 0.24)	0.001	0.15	(0.05; 0.24)	0.002	0.15	(0.06; 0.24)	0.002	0.12	(0.04; 0.21)	0.005	0.13	(0.04; 0.22)	0.006	0.11	(0.02; 0.19)	0.015
		Disadvantaged (51.5%)	0.36	(0.27; 0.44)	<0.001	0.34	(0.25; 0.42)	<0.001	0.34	(0.26; 0.43)	<0.001	0.24	(0.16; 0.32)	<0.001	0.30	(0.21; 0.38)	<0.001	0.19	(0.10; 0.27)	<0.001
**Whitehall (N=5114)**	Advantaged (51.2%)	Middle (31.8%)	0.16	(0.08; 0.23)	0.00003	0.16	(0.08; 0.23)	<0.001	0.12	(0.04; 0.19)	0.002	0.12	(0.05; 0.19)	0.001	0.14	(0.07; 0.22)	<0.001	0.07	(0; 0.14)	0.05
		Disadvantaged (17.0%)	0.37	(0.27; 0.47)	<0.001	0.37	(0.27; 0.47)	<0.001	0.31	(0.21; 0.41)	<0.001	0.27	(0.18; 0.37)	<0.001	0.32	0.22; 0.43)	<0.001	0.17	(0.07; 0.27)	0.001
**ELSA (N=5198)**	Advantaged (35.8%)	Middle (34.7%)	0.13	(0.06; 0.20)	0.0004	0.11	(0.04; 0.18)	0.004	0.11	(0.04; 0.18)	0.002	0.11	(0.05; 0.18)	0.001	0.11	(0.04; 0.18)	0.002	0.07	(-0.002; 0.13)	0.058
		Disadvantaged (29.5%)	0.29	(0.22; 0.37)	<0.001	0.25	(0.18; 0.33)	<0.001	0.25	(0.18; 0.33)	<0.001	0.24	(0.17; 0.31)	<0.001	0.25	(0.18; 0.33)	<0.001	0.14	(0.07; 0.21)	<0.001
**NCDS (N=5309)**	Advantaged (33.0%)	Middle (31.8%)	-0.03	(-0.11; 0.05)	0.488	-0.03	(-0.12; 0.05)	0.417	-0.04	(-0.12; 0.05)	0.378	-0.02	(-0.09; 0.06)	0.695	-			-0.03	(-0.10; 0.05)	0.499
		Disadvantaged (35.2%)	0.14	(0.06; 0.21)	<0.001	0.12	(0.04; 0.19)	0.003	0.10	(0.02; 0.17)	0.014	0.10	(0.03; 0.17)	0.006				0.04	(-0.03; 0.11)	0.248

Model 1 is adjusted for age and sex (except for NCDS, sex only)
Meta-analysis with random effect on 23008 participants (except for model 1 + sedentary where N = 17699)

Supplementary Table 8. Life course multiple regression analyses of SEP with CRP at baseline in 6 cohorts from the Lifepath project.

			Model A			Model B			Model C			Model D			Fully adjusted model		
			β	95% CI	P-value	β	95% CI	P-value	β	95% CI	P-value	β	95% CI	P-value	β	95% CI	P-value
**EPIC-Italy (N=2064)**	**Father's occupation**	Middle	0.29	(0.06; 0.51)	0.012	0.24	(0.01; 0.47)	0.038	0.28	(0.06; 0.51)	0.014	0.25	(0.02; 0.48)	0.033	0.20	(-0.02; 0.41)	0.068
		Disadvantaged	0.31	(0.09; 0.53)	0.005	0.25	(0.02; 0.47)	0.035	0.31	(0.08; 0.53)	0.008	0.26	(0.03; 0.49)	0.029	0.23	(0.02; 0.45)	0.034
	**Educational level**	Medium				0.09	(-0.08; 0.27)	0.300	-	-	-	0.10	(-0.08; 0.28)	0.276	0.08	(-0.09; 0.25)	0.352
		Low				0.16	(-0.01; 0.32)	0.064	-	-	-	0.18	(0.003; 0.36)	0.046	0.07	(-0.10; 0.24)	0.422
	**Last occupation**	Middle				-	-	-	-0.01	(-0.22; 0.20)	0.930	-0.03	(-0.24; 0.17)	0.748	0.01	(-0.18; 0.21)	0.898
		Disadvantaged				-	-	-	0.01	(-0.21; 0.22)	0.953	-0.07	(-0.30; 0.15)	0.531	-0.06	(-0.27; 0.15)	0.596
**NCDS (N=5309)**	**Father's occupation**	Middle	-0.08	(-0.23; 0.07)	0.273	-0.13	(-0.28; 0.02)	0.078	-0.09	(-0.24; 0.05)	0.216	-0.14	(-0.28; 0.01)	0.072	-0.16	(-0.30; -0.02)	0.026
		Disadvantaged	0.23	(0.10; 0.36)	0.001	0.12	(-0.02; 0.25)	0.084	0.21	(0.08; 0.34)	0.002	0.12	(-0.02; 0.25)	0.087	0.03	(-0.10; 0.16)	0.632
	**Educational level**	Medium				0.19	(0.04; 0.35)	0.016	-	-	-	0.20	(0.04; 0.35)	0.014	0.12	(-0.02; 0.27)	0.098
		Low				0.34	(0.25; 0.42)	<0.001	-	-	-	0.34	(0.25; 0.43)	<0.001	0.21	(0.13; 0.29)	<0.001
	**Last occupation**	Middle				-	-	-	-0.06	(-0.14; 0.03)	0.184	-0.10	(-0.19; -0.02)	0.014	-0.07	(-0.15; 0.004)	0.065
		Disadvantaged				-	-	-	0.09	(0.01; 0.17)	0.025	-0.01	(-0.09; 0.07)	0.815	-0.04	(-0.12; 0.03)	0.260
**Skipogh (N = 591)**	**Father's occupation**	Middle	0.27	(0.02; 0.51)	0.034	0.21	(-0.04; 0.45)	0.108	0.26	(0.01; 0.51)	0.039	0.21	(-0.04; 0.46)	0.095	0.20	(-0.04; 0.44)	0.102
		Disadvantaged	0.15	(-0.09; 0.39)	0.227	0.03	(-0.22; 0.28)	0.795	0.13	(-0.12; 0.37)	0.303	0.04	(-0.21; 0.29)	0.755	0.01	(-0.23; 0.26)	0.923
	**Educational level**	Medium				0.06	(-0.21; 0.32)	0.670	-	-	-	0.13	(-0.16; 0.41)	0.384	0.08	(-0.20; 0.35)	0.594
		Low				0.33	(0.08; 0.59)	0.012	-	-	-	0.41	(0.11; 0.71)	0.008	0.24	(-0.06; 0.54)	0.111
	**Last occupation**	Middle				-	-	-	-0.09	(-0.36; 0.19)	0.537	-0.20	(-0.49; 0.09)	0.179	-0.21	(-0.49; 0.08)	0.151
		Disadvantaged				-	-	-	0.07	(-0.19; 0.32)	0.620	-0.16	(-0.45; 0.14)	0.310	-0.16	(-0.46; 0.13)	0.270
**Whitehall (N = 5114)**	**Father's occupation**	Middle	0.04	(-0.08; 0.15)	0.564	0.01	(-0.11; 0.13)	0.853	0.01	(-0.11; 0.13)	0.866	0.00	(-0.12; 0.12)	0.996	-0.01	(-0.12; 0.10)	0.866
		Disadvantaged	0.14	(0.03; 0.25)	0.016	0.08	(-0.03; 0.20)	0.152	0.08	(-0.03; 0.20)	0.141	0.06	(-0.05; 0.18)	0.283	0.02	(-0.08; 0.13)	0.675
	**Educational level**	Medium				0.08	(-0.01; 0.16)	0.065	-	-	-	0.05	(-0.04; 0.13)	0.254	0.00	(-0.08; 0.08)	0.992
		Low				0.17	(0.09; 0.25)	<0.001	-	-	-	0.09	(0.001; 0.17)	0.048	0.01	(-0.07; 0.09)	0.739
	**Last occupation**	Middle				-	-	-	0.15	(0.08; 0.22)	<0.001	0.13	(0.05; 0.21)	0.001	0.06	(-0.01; 0.14)	0.085
		Disadvantaged				-	-	-	0.35	(0.25; 0.45)	<0.001	0.32	(0.21; 0.43)	<0.001	0.15	(0.05; 0.26)	0.004

Supplementary Table 9. Multiple regression analyses of participant's educational attainment with CRP at baseline in 6 cohorts from the Lifepath project after exclusion of participants with CRP ≥ 10mg/L.

			Model 1					Model 1 + Alcohol					Model 1 + Smoking					Model 1 + BMI					Model 1 + Sedentary					Model 2				
	Ref	Level	B	CI	P-value	I^2	P_H	B	CI	P-value	I^2	P_H	B	CI	P-value	I^2	P_H	B	CI	P-value	I^2	P_H	B	CI	P-value	I^2	P_H	B	CI	P-value	I^2	P_H
**Skipogh (N=553)**	High	Medium	0.01	(-0.21; 0.23)	0.934	-	-	0.001	(-0.22; 0.22)	0.994	-	-	0.01	(-0.21; 0.23)	0.936	-	-	-0.04	(-0.25; 0.17)	0.691	-	-	0.01	(-0.21; 0.23)	0.931	-	-	-0.05	(-0.26; 0.16)	0.647	-	-
		Low	0.10	(-0.10; 0.31)	0.320	-	-	0.10	(-0.11; 0.30)	0.356	-	-	0.11	(-0.10; 0.31)	0.314	-	-	-0.02	(-0.22; 0.19)	0.877	-	-	0.09	(-0.12; 0.30)	0.388	-	-	-0.02	(-0.22; 0.19)	0.868	-	-
**EPIC-Italy (N=2011)**	High	Medium	0.13	(-0.04; 0.29)	0.141	-	-	0.12	(-0.04; 0.29)	0.147	-	-	0.13	(-0.04; 0.30)	0.127	-	-	0.10	(-0.06; 0.25)	0.234	-	-	0.12	(-0.04; 0.29)	0.147	-	-	0.10	(-0.06; 0.26)	0.213	-	-
		Low	0.22	(0.07; 0.37)	0.004	-	-	0.22	(0.07; 0.37)	0.004	-	-	0.21	(0.06; 0.36)	0.005	-	-	0.11	(-0.03; 0.25)	0.128	-	-	0.22	(0.07; 0.37)	0.005	-	-	0.10	(-0.04; 0.24)	0.174	-	-
**CoLaus (N=4572)**	High	Medium	0.08	(-0.01; 0.17)	0.086	-	-	0.08	(-0.01; 0.17)	0.077	-	-	0.08	(-0.01; 0.17)	0.080	-	-	0.03	(-0.05; 0.12)	0.440	-	-	0.08	(-0.01; 0.17)	0.081	-	-	0.04	(-0.05; 0.12)	0.383	-	-
		Low	0.25	(0.19; 0.31)	<0.001	-	-	0.24	(0.18; 0.30)	<0.001	-	-	0.24	(0.18; 0.30)	<0.001	-	-	0.14	(0.08; 0.19)	<0.001	-	-	0.21	(0.14; 0.27)	<0.001	-	-	0.09	(0.03; 0.15)	0.002	-	-
**Whitehall (N=5024)**	High	Medium	0.10	(0.02; 0.18)	0.011	-	-	0.10	(0.02; 0.18)	0.010	-	-	0.08	(-0.002; 0.15)	0.057	-	-	0.05	(-0.02; 0.12)	0.175	-	-	0.10	(0.02; 0.18)	0.010	-	-	0.03	(-0.04; 0.10)	0.416	-	-
		Low	0.19	(0.12; 0.26)	<0.001	-	-	0.19	(0.12; 0.26)	<0.001	-	-	0.14	(0.07; 0.21)	<0.001	-	-	0.11	(0.04; 0.18)	0.002	-	-	0.19	(0.11; 0.26)	<0.001	-	-	0.06	(-0.01; 0.13)	0.091	-	-
**ELSA (N=4796)**	High	Medium	0.24	(0.16; 0.33)	<0.001	-	-	0.23	(0.15; 0.32)	<0.001	-	-	0.23	(0.14; 0.32)	<0.001	-	-	0.19	(0.10; 0.27)	<0.001	-	-	0.23	(0.14; 0.32)	<0.001	-	-	0.16	(0.07; 0.24)	<0.001	-	-
		Low	0.35	(0.27; 0.43)	<0.001	-	-	0.32	(0.25; 0.40)	<0.001	-	-	0.32	(0.24; 0.40)	<0.001	-	-	0.28	(0.20; 0.35)	<0.001	-	-	0.32	(0.24; 0.39)	<0.001	-	-	0.21	(0.13; 0.28)	<0.001	-	-
**NCDS (N=5182)**	High	Medium	0.19	(0.04; 0.33)	0.013	-	-	0.18	(0.03; 0.32)	0.020	-	-	0.16	(0.02; 0.31)	0.028	-	-	0.14	(0.00; 0.28)	0.049	-	-	-	-	-	-	-	0.11	(-0.03; 0.25)	0.128	-	-
		Low	0.36	(0.29; 0.44)	<0.001	-	-	0.35	(0.27; 0.42)	<0.001	-	-	0.32	(0.25; 0.40)	<0.001	-	-	0.27	(0.19; 0.34)	<0.001	-	-	-	-	-	-	-	0.21	(0.14; 0.28)	<0.001	-	-
**Meta-analyse (N= 22138)**	High	Medium	0.13	(0.07; 0.20)	<0.001	50.0	0.077	0.13	(0.07; 0.20)	<0.001	44.4	0.118	0.12	(0.06; 0.19)	<0.001	48.3	0.090	0.09	(0.02; 0.15)	0.007	50.2	0.073	0.12	(0.05; 0.19)	0.001	50.2	0.098	0.07	(0.02; 0.13)	0.011	37.1	0.183
		Low	0.26	(0.19; 0.34)	<0.001	72.3	0.004	0.25	(0.19; 0.32)	<0.001	65.4	0.014	0.24	(0.17; 0.31)	<0.001	71.2	0.004	0.17	(0.09; 0.24)	<0.001	79.2	<0.001	0.22	(0.16; 0.28)	<0.001	52.8	0.080	0.12	(0.06; 0.19)	<0.001	68.9	0.006
**Meta-analyse in Men (N= 12391)**	High	Medium	0.14	(0.09; 0.20)	<0.001	0.02	0.346	0.14	(0.09; 0.20)	<0.001	0.001	0.407	0.13	(0.07; 0.18)	<0.001	0.132	0.406	0.10	(0.04; 0.15)	<0.001	4.7	0.308	0.13	(0.07; 0.19)	<0.001	0	0.446	0.08	(0.03; 0.13)	0.002	0	0.514
		Low	0.25	(0.19; 0.31)	<0.001	36.4	0.076	0.25	(0.20; 0.29)	<0.001	6.3	0.141	0.20	(0.14; 0.26)	<0.001	40.6	0.08	0.17	(0.09; 0.25)	<0.001	63.8	0.021	0.21	(0.16; 0.26)	<0.001	0.056	0.307	0.12	(0.07; 0.17)	<0.001	20.8	0.113
**Meta-analyse in Women (N= 9747)**	High	Medium	0.13	(0.02; 0.25)	0.025	54.8	0.046	0.12	(0.01; 0.23)	0.029	51.1	0.071	0.13	(0.01; 0.24)	0.030	54.0	0.053	0.08	(-0.03; 0.18)	0.161	53.1	0.061	0.12	(-0.001; 0.25)	0.052	55.8	0.058	0.06	(-0.04; 0.16)	0.239	46.9	0.111
		Low	0.30	(0.19; 0.41)	<0.001	69.7	0.007	0.28	(0.17; 0.39)	<0.001	69.1	0.008	0.29	(0.17; 0.40)	<0.001	70.6	0.006	0.17	(0.06; 0.28)	0.002	73.3	0.002	0.25	(0.14; 0.36)	<0.001	60.5	0.045	0.14	(0.03; 0.24)	0.014	71.8	0.004

Model 1 is adjusted for age and sex (except for NCDS, sex only)
Meta-analysis with random effect on a total of 22138 participants (except for model 1 + sedentary where N = 16956)

Supplementary Table 10. Multiple regression analyses of participant's educational attainment with CRP at first follow up available only in a subset of 4 cohorts from the Lifepath project.

			Model 1					Model 1 + Alcohol					Model 1 + Smoking					Model 1 + BMI					Model 1 + Sedentary					Model 2				
**First follow-up**	**Ref.**	Level	B	CI	P-value	I^2	P_H	B	CI	P-value	I^2	P_H	B	CI	P-value	I^2	P_H	B	CI	P-value	I^2	P_H	B	CI	P-value	I^2	P_H	B	CI	P-value	I^2	P_H
Skipogh (N=232)	High	Medium	0.28	(-0.05; 0.61)	0.103			0.25	(-0.08; 0.59)	0.135			0.27	(-0.06; 0.60)	0.112			0.26	(-0.07; 0.59)	0.118			0.28	(-0.05; 0.61)	0.097			0.24	(-0.09; 0.57)	0.156		
		Low	0.37	(0.07; 0.67)	0.017			0.34	(0.04; 0.65)	0.029			0.38	(0.07; 0.68)	0.016			0.39	(0.09; 0.70)	0.013			0.33	(0.02; 0.63)	0.038			0.35	(0.04; 0.66)	0.030		
CoLaus (N=3636)	High	Medium	0.12	(0.02; 0.22)	0.022			0.12	(0.02; 0.23)	0.019			0.12	(0.02; 0.23)	0.019			0.08	(-0.02; 0.18)	0.106			0.12	(0.02; 0.23)	0.019			0.09	(-0.01; 0.19)	0.070		
		Low	0.26	(0.19; 0.34)	<0.001			0.25	(0.17; 0.32)	<0.001			0.25	(0.18; 0.33)	<0.001			0.17	(0.10; 0.25)	<0.001			0.22	(0.15; 0.30)	<0.001			0.13	(0.06; 0.21)	<0.001		
Whitehall (N=4027)	High	Medium	0.09	(-0.003; 0.18)	0.057			0.09	(0.00; 0.18)	0.051			0.06	(-0.03; 0.15)	0.189			0.05	(-0.03; 0.14)	0.238			0.09	(0.00; 0.18)	0.050			0.03	(-0.05; 0.12)	0.481		
		Low	0.18	(0.09; 0.26)	<0.001			0.18	(0.09; 0.26)	<0.001			0.13	(0.04; 0.21)	0.004			0.12	(0.04; 0.20)	0.005			0.18	(0.09; 0.26)	<0.001			0.06	(-0.02; 0.14)	0.132		
ELSA (N=3532)	High	Medium	0.26	(0.14; 0.37)	<0.001			0.24	(0.13; 0.36)	<0.001			0.24	(0.12; 0.36)	<0.001			0.21	(0.10; 0.32)	<0.001			0.24	(0.12; 0.35)	<0.001			0.17	(0.06; 0.28)	0.002		
		Low	0.38	(0.28; 0.48)	<0.001			0.34	(0.24; 0.45)	<0.001			0.34	(0.24; 0.45)	<0.001			0.30	(0.20; 0.40)	<0.001			0.33	(0.23; 0.43)	<0.001			0.23	(0.13; 0.33)	<0.001		
Meta-analyse (N=11427)	High	Medium	0.16	(0.07; 0.25)	0.001	53.1	0.112	0.15	(0.07; 0.24)	<0.001	43.3	0.186	0.15	(0.05; 0.24)	0.003	56.2	0.088	0.12	(0.03; 0.20)	0.008	50.7	0.122	0.15	(0.07; 0.23)	<0.001	39.6	0.200	0.10	(0.02; 0.18)	0.012	41.0	0.187
		Low	0.28	(0.18; 0.38)	<0.001	69.3	0.029	0.26	(0.17; 0.34)	<0.001	57.0	0.098	0.25	(0.14; 0.36)	<0.001	75.4	0.009	0.21	(0.11; 0.31)	<0.001	72.6	0.020	0.24	(0.17; 0.32)	<0.001	51.4	0.138	0.15	(0.06; 0.24)	0.001	64.4	0.046
**Second follow-up**																																
Whitehall (N=3927)	High	Medium	0.14	(0.06; 0.23)	0.001			0.15	(0.06; 0.23)	0.001			0.12	(0.03; 0.20)	0.007			0.11	(0.02; 0.19)	0.011			0.15	(0.06; 0.23)	0.001			0.09	(0.004; 0.17)	0.041		
		Low	0.17	(0.09; 0.25)	<0.001			0.16	(0.08; 0.24)	<0.001			0.13	(0.05; 0.21)	0.002			0.11	(0.03; 0.19)	0.007			0.17	(0.08; 0.25)	<0.001			0.07	(-0.01; 0.14)	0.101		
ELSA (N=3046)	High	Medium	0.27	(0.14; 0.39)	<0.001			0.25	(0.13; 0.37)	<0.001			0.24	(0.12; 0.36)	<0.001			0.23	(0.12; 0.35)	<0.001			0.24	(0.12; 0.36)	<0.001			0.18	(0.07; 0.30)	0.002		
		Low	0.38	(0.28; 0.49)	<0.001			0.34	(0.23; 0.45)	<0.001			0.34	(0.24; 0.45)	<0.001			0.32	(0.22; 0.43)	<0.001			0.34	(0.23; 0.45)	<0.001			0.23	(0.12; 0.33)	<0.001		

Model 1 is adjusted for age and sex

Supplementary Table 11. Multiple regression analyses using the disadvantaged/low group as a reference for (A) father's occupational position in 4 cohorts (B) participant's educational attainment and (C) participant's last occupation with CRP at baseline in 6 cohorts from the Lifepath project.

		**(A) Father's occupational position (N=13078)†**		**(B) Participant's educational attainment (N=23008)‡**		**(C) Participant's last occupation (N=23008)‡**	
	Reference	Disadvantaged (65.3%)		Low (58.3%)		Disadvantaged (34.4%)	
	Level	Middle (26.6%)	Advantaged (8.0%)	Medium (17.9%)	High (23.8%)	Middle (34.0%)	Advantaged (31.6%)
**Model 1***	β (95% CI)	-0.09 (-0.26; 0.08)	-0.19 (-0.27; -0.11)	-0.14 (-0.19; -0.10)	-0.30 (-0.38; -0.22)	-0.16 (-0.21; -0.11)	-0.24 (-0.35; -0.14)
	P-value	0.277	<0.001	<0.001	<0.001	<0.001	<0.001
	I^2	91.3	1.3	0.05	75.6	42.2	81.4
	P_H	<0.001	0.466	0.514	0.001	0.140	<0.001
**Model 1* + Alcohol**	β (95% CI)	-0.09 (-0.26; 0.07)	-0.18 (-0.26; -0.11)	-0.13 (-0.17; -0.09)	-0.29 (-0.36; -0.21)	-0.15 (-0.20; -0.10)	-0.22 (-0.33; -0.12)
	P-val	0.279	<0.001	<0.001	<0.001	<0.001	<0.001
	I^2	90.9	0	0.05	68.5	35.4	80.9
	P_H	<0.001	0.518	0.616	0.008	0.180	<0.001
**Model 1* + Smoking**	β (95% CI)	-0.09 (-0.24; 0.07)	-0.18 (-0.25; -0.10)	-0.13 (-0.17; -0.08)	-0.27 (-0.35; -0.19)	-0.14 (-0.19; -0.09)	-0.21 (-0.31; -0.12)
	P-value	0.283	<0.001	<0.001	<0.001	<0.001	<0.001
	I^2	90.0	0	0.01	74.6	40.2	79.1
	P_H	<0.001	0.452	0.445	0.001	0.156	<0.001
**Model 1* + BMI**	β (95% CI)	-0.06 (-0.21; 0.10)	-0.10 (-0.17; -0.03)	-0.09 (-0.14; -0.05)	-0.19 (-0.28; -0.11)	-0.10 (-0.15; -0.05)	-0.17 (-0.25; -0.09)
	P-value	0.465	0.005	<0.001	<0.001	<0.001	<0.001
	I^2	90.5	0	21.7	79.3	52.1	73.3
	P_H	<0.001	0.451	0.301	<0.001	0.084	0.004
**Model 1*+ Sedentary**	β (95% CI)	-0.04 (-0.13; 0.05)	-0.17 (-0.26; -0.08)	-0.12 (-0.16; -0.08)	-0.25 (-0.32; -0.18)	-0.14 (-0.18; -0.10)	-0.26 (-0.32; -0.21)
	P-value	0.366	<0.001	<0.001	<0.001	<0.001	<0.001
	I^2	50.4	0	0	60.4	9.5	18.5
	P_H	0.120	0.381	0.678	0.048	0.349	0.099
**Model 2¤**	β (95% CI)	-0.05 (-0.19; 0.09)	-0.08 (-0.15; -0.01)	-0.06 (-0.09; -0.02)	-0.14 (-0.21; -0.07)	-0.06 (-0.10; -0.03)	-0.11 (-0.17; -0.05)
	P-value	0.476	0.021	0.005	<0.001	<0.001	<0.001
	I^2	88.0	0	0	67.2	0	51.4
	P_H	<0.001	0.458	0.517	0.009	0.335	0.065

†Except for model 1 + sedentary where N = 7769

‡Except for model 1 + sedentary where N = 17699

*Model 1 adjusted for age and sex

¤Model 2 controlled for age, sex and additionally alcohol, smoking, BMI and sedentary

Abbreviations: CI, confidence interval; I^2, percentage of between study heterogeneity; P_H, P-value of heterogeneity test; BMI, body mass index.

Supplementary Table 12. Multiple regression analyses using SEP in 2 classes for (A) father's occupational position in 4 cohorts (B) participant's last occupation with CRP at baseline in 6 cohorts from the Lifepath project.

		**(A) Father's occupational position (N=13078)†**	**(B) Participant's last occupation (N=23008)‡**
	Reference	Advantaged (43.2%)	Advantaged (70.8%)
	Level	Disadvantaged (56.8%)	Disadvantaged (29.2%)
**Model 1***	β (95% CI)	0.16 (0.03; 0.29)	0.17 (0.10; 0.24)
	P-value	0.013	<0.001
	I^2	87.4	74.9
	P_H	<0.001	0.003
**Model 1* + Alcohol**	β (95% CI)	0.16 (0.03; 0.28)	0.16 (0.09; 0.23)
	P-val	0.012	<0.001
	I^2	86.5	70.7
	P_H	<0.001	0.011
**Model 1* + Smoking**	β (95% CI)	0.16 (0.03; 0.28)	0.15 (0.09; 0.21)
	P-value	0.012	<0.001
	I^2	86.0	62.7
	P_H	<0.001	0.034
**Model 1* + BMI**	β (95% CI)	0.09 (-0.02; 0.19)	0.11 (0.03; 0.18)
	P-value	0.100	0.005
	I^2	82.4	78.3
	P_H	<0.001	0.001
**Model 1*+ Sedentary**	β (95% CI)	0.10 (-0.004; 0.21)	0.15 (0.08; 0.23)
	P-value	0.059	<0.001
	I^2	64.0	68.2
	P_H	0.091	0.019
**Model 2¤**	β (95% CI)	0.08 (-0.02; 0.17)	0.06 (0.02; 0.11)
	P-value	0.105	0.003
	I^2	77.5	36.2
	P_H	0.002	0.164

†Except for model 1 + sedentary where N = 7769

‡Except for model 1 + sedentary where N = 17699

*Model 1 adjusted for age and sex

¤Model 2 controlled for age, sex and additionally alcohol, smoking, BMI and sedentary

Abbreviations: CI, confidence interval; I^2, percentage of between study heterogeneity; P_H, P-value of heterogeneity test; BMI, body mass index.

Supplementary Table 13. Additional information about Lifepath cohorts

	Design	Date of recruitment	Data collection	CRP measure	First CRP used in the study	Ethics statement
**EPIC-Italy**	Prospective cohort	1993 - 1998	Self-administered dietary and lifestyle questionnaire<br>Standardized blood sample and anthropometric measures	HS CRP was measured in plasma, by latex particle-enhanced immunoturbidimetric assay (IL Coagulation Systems on ACL9000)	At baseline, between 1993 - 1998	Approved by the ethical review boards of the International Agency for Research on Cancer, and of the collaborating institutions responsible for subject recruitment in each of the EPIC recruitment centres.
**CoLaus**	Population-based cross-sectional study	2003 - 2006	Demographic data, socio-economic and marital status, and several lifestyle factors questionnaire.<br>Standardized anthropometric measure	HS-CRP (hs-CRP, mg/L) was assessed by immunoassay and latex HS (IMMULITE 1000-High, Diagnostic Products Corporation, Los Angeles, CA, USA)	At baseline, between 2003 - 2006	Approved by the Institutional Ethics Committee of the University of Lausanne (Switzerland)
**Skipogh**	Longitudinal family-based study	2009 - 2013	Participants come to the study center an overnight fast. They are welcomed by one of the medical assistants or study nurses. Total duration of the 'check-up' is 2h-2h30.<br><br>A questionnaire includes questions on previous medical history, medication intake, professional life, and life habits (alimentation, physical activity) was given.	HS-CRP (hs-CRP, mg/L) was assessed by immunoassay and latex HS (Roche Diagnostics, CH)	At baseline, between 2009 - 2013	Approved by the Human Research Ethics Committee, Lausanne University Hospital and University of Lausanne (Lausanne, Switzerland), by the Ethics Committee for the Research on Human Beings, Geneva University Hospitals (Geneva, Switzerland), and by the Ethics Committee of the Canton of Bern, (Bern, Switzerland).
**Whitehall**	Prospective occupational cohort	From 1985 (12 waves)	Clinical examination and a self-administered questionnaire containing sections on demographic characteristics, health, lifestyle factors, work characteristics, social support, and life events.	CRP was measured using a high-sensitivity immunonephelometric assay in a BN ProSpec nephelometer (Dade Behring, Milton Keynes, Bucks, UK).	In the third wave, between 1991 - 1994	The University College London ethics committee approved this study
**ELSA**	English longitudinal study of aging	Recruited from Health Survey for England in 1998, 1999, 2001	Face-to-face interview (a computer-assisted personal interview followed by a self-completion questionnaire) every two years of the study and a nurse assessment every four years (measurements of physical function, anthropometric measurements and collection of blood samples).	CRP was measured using the N Latex CRP mono immunoassay on the Behring Nephelometer II analyzer.	In the second wave, between 2004 - 2005	Ethical consent has been obtained for all waves and components of ELSA, according to the ethical approval system in operation at the time.
**NCDS**	National longitudinal birth cohort study	Babies born in England, Scotland and Wales in one week of 1958<br>(9 swepts and new survey in 2018)	Information was mostly reported by the participant, and measurements were collected for the biomedical survey at 45y.	CRP was measured on citrated plasma by high-sensitivity nephelometric analysis of latex particles coated with CRP-monoclonal antibodies. (BN ProSpec protein analyzer, Dade Behring, Marburg, Germany.)		Ethical approval for NCDS was granted by the London Multi-Centre Research Ethics Committee.

Supplementary Table 14: Multiple regression analyses testing the interaction between SEP and gender for (A) father's occupational position in 4 cohorts (B) participant's last occupation with CRP at baseline in 6 cohorts from the Lifepath project.

			**(A) Father occupational position**											
			**Model 1**		**Model 1 + Alcohol**		**Model 1 + Smoking**		**Model 1 + BMI**		**Model 1 + Sedentary**		**Fully adjusted**	
	Ref.	Levels	β (95% CI)	pvalue	β (95% CI)	pvalue	β (95% CI)	pvalue	β (95% CI)	pvalue	β (95% CI)	pvalue	β (95% CI)	pvalue
**Skipogh**	High:Men	Medium:Women	0.082 (-0.412; 0.576)	0.74	0.068 (-0.427; 0.564)	0.79	0.083 (-0.412; 0.579)	0.74	-0.035 (-0.51; 0.44)	0.88	0.069 (-0.424; 0.562)	0.78	-0.049 (-0.528; 0.429)	0.84
		Low:Women	-0.03 (-0.51; 0.449)	0.90	-0.035 (-0.515; 0.446)	0.89	-0.028 (-0.51; 0.454)	0.91	-0.139 (-0.598; 0.319)	0.55	-0.029 (-0.508; 0.449)	0.90	-0.138 (-0.599; 0.324)	0.56
**EPIC-Italy**	High:Men	Medium:Women	0.265 (-0.183; 0.714)	0.246	0.268 (-0.181; 0.716)	0.24	0.267 (-0.178; 0.712)	0.24	0.178 (-0.247; 0.602)	0.41	0.254 (-0.193; 0.701)	0.27	0.176 (-0.245; 0.596)	0.41
		Low:Women	0.315 (-0.127; 0.757)	0.162	0.319 (-0.123; 0.761)	0.16	0.342 (-0.097; 0.781)	0.13	0.159 (-0.26; 0.577)	0.46	0.32 (-0.121; 0.76)	0.16	0.193 (-0.222; 0.607)	0.36
**Whitehall**	High:Men	Medium:Women	0.151 (-0.109; 0.412)	0.255	0.143 (-0.117; 0.403)	0.28	0.162 (-0.096; 0.42)	0.22	0.08 (-0.166; 0.326)	0.52	0.129 (-0.131; 0.388)	0.33	0.068 (-0.175; 0.311)	0.58
		Low:Women	0.192 (-0.051; 0.434)	0.121	0.181 (-0.061; 0.423)	0.14	0.163 (-0.077; 0.404)	0.18	0.066 (-0.163; 0.296)	0.57	0.167 (-0.074; 0.409)	0.17	0.018 (-0.209; 0.245)	0.88
**NCDS**	High:Men	Medium:Women	-0.008 (-0.306; 0.289)	0.956	-0.017 (-0.314; 0.28)	0.91	-0.022 (-0.318; 0.274)	0.89	0.065 (-0.216; 0.345)	0.65	-	-	0.042 (-0.236; 0.32)	0.77
		Low:Women	0.13 (-0.134; 0.394)	0.334	0.123 (-0.14; 0.387)	0.36	0.112 (-0.151; 0.375)	0.40	0.172 (-0.077; 0.421)	0.18	-	-	0.144 (-0.103; 0.391)	0.25
			**(B) Participant's educational attainment**											
			**Model 1**		**Model 1 + Alcohol**		**Model 1 + Smoking**		**Model 1 + BMI**		**Model 1 + Sedentary**		**Fully adjusted**	
	Ref.	Levels	β (95% CI)	pvalue	β (95% CI)	pvalue	β (95% CI)	pvalue	β (95% CI)	pvalue	β (95% CI)	pvalue	β (95% CI)	pvalue
**Skipogh**	High:Men	Medium:Women	-0.011 (-0.538; 0.516)	0.967	-0.015 (-0.543; 0.514)	0.96	-0.007 (-0.536; 0.522)	0.98	0.01 (-0.496; 0.516)	0.97	-0.008 (-0.534; 0.518)	0.98	0.023 (-0.487; 0.533)	0.93
		Low:Women	0.07 (-0.423; 0.563)	0.780	0.08 (-0.416; 0.576)	0.75	0.074 (-0.421; 0.568)	0.77	0.058 (-0.417; 0.533)	0.81	0.093 (-0.4; 0.586)	0.71	0.086 (-0.394; 0.566)	0.72
**EPIC-Italy**	High:Men	Medium:Women	0.417 (0.069; 0.766)	0.019	0.42 (0.07; 0.769)	0.02	0.437 (0.09; 0.783)	0.01	0.338 (0.008; 0.669)	0.05	0.391 (0.043; 0.739)	0.03	0.348 (0.02; 0.676)	0.04
		Low:Women	0.412 (0.102; 0.721)	0.009	0.416 (0.106; 0.727)	0.01	0.465 (0.157; 0.774)	0.00	0.334 (0.04; 0.628)	0.03	0.387 (0.078; 0.696)	0.01	0.387 (0.094; 0.679)	0.01
**Colaus**	High:Men	Medium:Women	-0.123 (-0.315; 0.069)	0.208	-0.124 (-0.316; 0.068)	0.21	-0.139 (-0.33; 0.053)	0.16	-0.055 (-0.235; 0.124)	0.55	-0.117 (-0.308; 0.074)	0.23	-0.073 (-0.251; 0.106)	0.42
		Low:Women	-0.082 (-0.215; 0.052)	0.230	-0.093 (-0.227; 0.04)	0.17	-0.08 (-0.214; 0.053)	0.24	-0.086 (-0.211; 0.039)	0.18	-0.072 (-0.205; 0.061)	0.29	-0.085 (-0.209; 0.039)	0.18
**Whitehall**	High:Men	Medium:Women	-0.132 (-0.333; 0.07)	0.200	-0.118 (-0.319; 0.083)	0.25	-0.142 (-0.341; 0.058)	0.16	-0.16 (-0.35; 0.03)	0.10	-0.118 (-0.319; 0.082)	0.25	-0.154 (-0.342; 0.034)	0.11
		Low:Women	-0.019 (-0.19; 0.151)	0.827	-0.015 (-0.185; 0.156)	0.87	-0.025 (-0.194; 0.144)	0.77	-0.084 (-0.246; 0.077)	0.30	-0.022 (-0.192; 0.148)	0.80	-0.094 (-0.253; 0.066)	0.25
**ELSA**	High:Men	Medium:Women	0.043 (-0.164; 0.25)	0.687	0.033 (-0.174; 0.239)	0.76	0.035 (-0.171; 0.241)	0.74	0.019 (-0.178; 0.217)	0.85	0.028 (-0.177; 0.233)	0.79	-0.007 (-0.201; 0.187)	0.95
		Low:Women	0.04 (-0.139; 0.218)	0.665	0.033 (-0.146; 0.211)	0.72	0.047 (-0.13; 0.225)	0.60	-0.003 (-0.174; 0.168)	0.97	0.034 (-0.143; 0.212)	0.70	-0.005 (-0.173; 0.163)	0.96
**NCDS**	High:Men	Medium:Women	-0.103 (-0.417; 0.211)	0.519	-0.107 (-0.42; 0.207)	0.51	-0.098 (-0.41; 0.215)	0.54	-0.098 (-0.41; 0.215)	0.54	-	-	-0.077 (-0.371; 0.217)	0.61
		Low:Women	0.045 (-0.117; 0.208)	0.584	0.034 (-0.129; 0.196)	0.68	0.048 (-0.114; 0.21)	0.56	0.048 (-0.114; 0.21)	0.56	-	-	0.023 (-0.13; 0.175)	0.77
			**(C) Participant's last occupation**											
			**Model 1**		**Model 1 + Alcohol**		**Model 1 + Smoking**		**Model 1 + BMI**		**Model 1 + Sedentary**		**Fully adjusted**	
	Ref.	Levels	β (95% CI)	pvalue	β (95% CI)	pvalue	β (95% CI)	pvalue	β (95% CI)	pvalue	β (95% CI)	pvalue	β (95% CI)	pvalue
**Skipogh**	High:Men	Medium:Women	-0.161 (-0.716; 0.394)	0.570	-0.168 (-0.724; 0.388)	0.55	-0.162 (-0.719; 0.394)	0.57	-0.178 (-0.711; 0.354)	0.51	-0.132 (-0.686; 0.422)	0.64	-0.175 (-0.711; 0.36)	0.52
		Low:Women	0.294 (-0.23; 0.819)	0.272	0.287 (-0.238; 0.813)	0.28	0.295 (-0.231; 0.82)	0.27	0.181 (-0.323; 0.685)	0.48	0.322 (-0.201; 0.846)	0.23	0.188 (-0.319; 0.695)	0.47
**EPIC-Italy**	High:Men	Medium:Women	0.242 (-0.187; 0.671)	0.269	0.243 (-0.187; 0.672)	0.27	0.221 (-0.206; 0.648)	0.31	0.142 (-0.264; 0.549)	0.49	0.244 (-0.184; 0.672)	0.26	0.126 (-0.277; 0.529)	0.54
		Low:Women	0.308 (-0.125; 0.74)	0.163	0.311 (-0.123; 0.744)	0.16	0.338 (-0.092; 0.768)	0.12	0.141 (-0.269; 0.551)	0.50	0.306 (-0.125; 0.738)	0.16	0.183 (-0.224; 0.59)	0.38
**Colaus**	High:Men	Medium:Women	0.166 (-0.034; 0.365)	0.104	0.16 (-0.04; 0.36)	0.12	0.167 (-0.033; 0.366)	0.10	0.08 (-0.107; 0.267)	0.40	0.177 (-0.022; 0.376)	0.08	0.085 (-0.101; 0.271)	0.37
		Low:Women	0.108 (-0.081; 0.296)	0.263	0.094 (-0.094; 0.282)	0.33	0.111 (-0.078; 0.299)	0.25	0.049 (-0.127; 0.226)	0.58	0.124 (-0.064; 0.311)	0.20	0.054 (-0.121; 0.229)	0.55
**Whitehall**	High:Men	Medium:Women	0.145 (-0.034; 0.325)	0.113	0.156 (-0.024; 0.335)	0.09	0.166 (-0.012; 0.344)	0.07	0.089 (-0.081; 0.259)	0.30	0.147 (-0.032; 0.326)	0.11	0.112 (-0.056; 0.28)	0.19
		Low:Women	0.077 (-0.139; 0.293)	0.486	0.08 (-0.137; 0.296)	0.47	0.139 (-0.076; 0.354)	0.20	-0.023 (-0.228; 0.182)	0.83	0.11 (-0.107; 0.326)	0.32	0.054 (-0.15; 0.258)	0.61
**ELSA**	High:Men	Medium:Women	-0.169 (-0.312; -0.027)	0.020	-0.157 (-0.3; -0.015)	0.03	-0.158 (-0.299; -0.016)	0.03	-0.163 (-0.299; -0.027)	0.02	-0.141 (-0.282; 0.001)	0.05	-0.123 (-0.256; 0.011)	0.07
		Low:Women	-0.02 (-0.174; 0.133)	0.795	-0.027 (-0.18; 0.125)	0.73	-0.009 (-0.161; 0.143)	0.91	-0.047 (-0.193; 0.099)	0.53	-0.011 (-0.163; 0.14)	0.88	-0.031 (-0.174; 0.113)	0.68
**NCDS**	High:Men	Medium:Women	-0.171 (-0.342; 0)	0.050	-0.168 (-0.338; 0.003)	0.05	-0.169 (-0.339; 0.001)	0.05	-0.137 (-0.298; 0.023)	0.09	-	-	-0.133 (-0.292; 0.027)	0.10
		Low:Women	0.032 (-0.128; 0.191)	0.697	0.021 (-0.139; 0.18)	0.80	0.048 (-0.111; 0.206)	0.56	0.022 (-0.128; 0.172)	0.77	-	-	0.033 (-0.117; 0.182)	0.67

*Model 1 adjusted for age and sex

¤Model 2 controlled for age, sex and additionally alcohol, smoking, BMI and sedentary

Abbreviations: CI, confidence interval; BMI, body mass index.
