# Supplementary material for: Multi-cohort study identifies social determinants of systemic inflammation over the life course
Source: Nat Commun. 2019 Feb 15;10:773. doi: 10.1038/s41467-019-08732-x (PMC6377676; doi:10.1038/s41467-019-08732-x)
Supplement: Supplementary file 3 — Description of Additional Supplementary Files [file 41467_2019_8732_MOESM3_ESM.pdf]

Description of Additional Supplementary Files

File Name: Supplementary Data 1
Description: Table of descriptive statistics for each cohort.

File Name: Supplementary Data 2
Description: Description of each cohort by life course socioeconomic position
