# Supplementary material for: Multi-cohort study identifies social determinants of systemic inflammation over the life course
Source: Nat Commun. 2019 Feb 15;10:773. doi: 10.1038/s41467-019-08732-x (PMC6377676; doi:10.1038/s41467-019-08732-x)
Supplement: Supplementary file 6 — Reporting Summary [file 41467_2019_8732_MOESM6_ESM.pdf]

nature research

Corresponding author(s): Michelle Kelly Irving

Reporting Summary

Nature Research wishes to improve the reproducibility of the work that we publish. This form provides structure for consistency and transparency in reporting. For further information on Nature Research policies, see Authors & Referees and the Editorial Policy Checklist.

Statistical parameters

When statistical analyses are reported, confirm that the following items are present in the relevant location (e.g. figure legend, table legend, main text, or Methods section).

n/a	Confirmed	
☐	☒	The exact sample size (*n*) for each experimental group/condition, given as a discrete number and unit of measurement
☐	☒	An indication of whether measurements were taken from distinct samples or whether the same sample was measured repeatedly
☐	☒	The statistical test(s) used AND whether they are one- or two-sided<br>*Only common tests should be described solely by name; describe more complex techniques in the Methods section.*
☐	☒	A description of all covariates tested
☐	☒	A description of any assumptions or corrections, such as tests of normality and adjustment for multiple comparisons
☐	☒	A full description of the statistics including central tendency (e.g. means) or other basic estimates (e.g. regression coefficient) AND variation (e.g. standard deviation) or associated estimates of uncertainty (e.g. confidence intervals)
☐	☒	For null hypothesis testing, the test statistic (e.g. *F*, *t*, *r*) with confidence intervals, effect sizes, degrees of freedom and *P* value noted<br>*Give P values as exact values whenever suitable.*
☒	☐	For Bayesian analysis, information on the choice of priors and Markov chain Monte Carlo settings
☒	☐	For hierarchical and complex designs, identification of the appropriate level for tests and full reporting of outcomes
☐	☒	Estimates of effect sizes (e.g. Cohen's *d*, Pearson's *r*), indicating how they were calculated
☐	☒	Clearly defined error bars<br>*State explicitly what error bars represent (e.g. SD, SE, CI)*

Our web collection on statistics for biologists may be useful.

Software and code

Policy information about availability of computer code

Data collection	*Provide a description of all commercial, open source and custom code used to collect the data in this study, specifying the version used OR state that no software was used.*
Data analysis	Statistical analyses were performed using R version 3.5.0.

For manuscripts utilizing custom algorithms or software that are central to the research but not yet described in published literature, software must be made available to editors/reviewers upon request. We strongly encourage code deposition in a community repository (e.g. GitHub). See the Nature Research guidelines for submitting code & software for further information.

Data

Policy information about availability of data

All manuscripts must include a data availability statement. This statement should provide the following information, where applicable:

- Accession codes, unique identifiers, or web links for publicly available datasets
- A list of figures that have associated raw data
- A description of any restrictions on data availability

The data and computer code uses to support the findings of this study are available from the corresponding author upon reasonable request.

Field-specific reporting

Please select the best fit for your research. If you are not sure, read the appropriate sections before making your selection.

- [ ] Life sciences
- [x] Behavioural & social sciences
- [ ] Ecological, evolutionary & environmental sciences

For a reference copy of the document with all sections, see nature.com/authors/policies/ReportingSummary-flat.pdf

Behavioural & social sciences study design

All studies must disclose on these points even when the disclosure is negative.

Study description	Lifepath is a Horizon 2020 Research and Innovation Program European funded project which aims to investigate the biological pathways underlying social differences in healthy ageing. For this purpose, the project includes a consortium of eighteen cohort studies (child and adult) across different countries and time periods, with demographic, clinical, biological and socioeconomic data. Data harmonization was performed in order to merge and analyse the cohorts together.
Research sample	We selected six cohorts for which data on CRP, at least two measures of socioeconomic position at two different life course stages, and behaviours/BMI were available. Our panel of cohorts includes a subset of the Italian component of the European prospective investigation on nutrition and cancer study (EPIC-Italy); two cohorts from Switzerland: CoLaus and Skipogh; and three British cohorts: Whitehall II, the English longitudinal study of ageing (ELSA), and the National Child Development Study (NCDS).
Sampling strategy	In all of the six cohorts, biological measures was done in a sub-population of participants. We have focus for this study on these sub-populations and have selected only participants for whom CRP measure was available. Then we have included in our study population only participants with complete data on SEP and intermediate factors.
Data collection	- EPIC-Italy: Self-administered dietary and lifestyle questionnaire and standardized blood sample and anthropometric measures<br>- CoLaus: Demographic data, socio-economic and marital status, and several lifestyle factors questionnaire and standardized anthropometric measure<br>- Skipogh: Participants came to the study center an overnight fast and were welcomed by one of the medical assistants or study nurses. Total duration of the 'check-up' is 2h-2h30. A questionnaire includes questions on previous medical history, medication intake, professional life, and life habits (alimentation, physical activity) was given.<br>- Whitehall: Clinical examination and a self-administered questionnaire containing sections on demographic characteristics, health, lifestyle factors, work characteristics, social support, and life events.<br>- ELSA: Face-to-face interview (a computer-assisted personal interview followed by a self-completion questionnaire) every two years of the study and a nurse assessment every four years (measurements of physical function, anthropometric measurements and collection of blood samples).<br>- NCDS: Information was mostly reported by the participant, and measurements were collected for the biomedical survey at 45y.
Timing	Recruitement between:<br>- 1993/1998 for EPIC-Italy<br>- 2003/2006 for CoLaus<br>- 2009/2013 for Skipogh<br>- From 1985 for Whitehall<br>- 1998, 1999, 2001 for ELSA<br>- one week of 1958 for NCDS (birth cohort) - biomedical survey 2002-2004
Data exclusions	Participants without CRP measure or with missing data on socioeconomic position or intermediate factors.<br>Sensitivity analyses with exclusion of participants with CRP above 10mg/L.
Non-participation	*State how many participants dropped out/declined participation and the reason(s) given OR provide response rate OR state that no participants dropped out/declined participation.*
Randomization	NA

Reporting for specific materials, systems and methods

Materials & experimental systems

n/a	Involved in the study
☒	☐ Unique biological materials
☒	☐ Antibodies
☒	☐ Eukaryotic cell lines
☒	☐ Palaeontology
☒	☐ Animals and other organisms
☐	☒ Human research participants

Methods

n/a	Involved in the study
☒	☐ ChIP-seq
☒	☐ Flow cytometry
☒	☐ MRI-based neuroimaging

Human research participants

Policy information about studies involving human research participants

Population characteristics

See above.

Recruitment

Describe how participants were recruited. Outline any potential self-selection bias or other biases that may be present and how these are likely to impact results.
